# Local Anesthesia

**Published:** 1921-04

**Authors:** J. H. Anderson

**Affiliations:** England


### LOCAL ANESTHESIA

BY J. H. ANDERSON, L.D.S., ENGLAND

Local anesthesia, although useful and invaluable in particular cases, has by no means displaced nitrous oxide from the glorious pinnacle which it has occupied and still maintains in dental surgery. While nitrous oxide still remains a most useful general anesthetic, and has its uses where local anesthesia is inapplicable and undesirable, it can not be denied that the latter anesthesia has afforded us a great amount of comfort and confidence in its application in dental surgery for its special cases. From this it follows that local anesthesia is an invaluable help when used with discrimination.

Before proceeding further it is interesting first of all to refer briefly to the history and enumerate the different attempts which led up to the present stage of local anesthesia in the progress of its advance.

The first attempts at local anesthesia were made with the ether spray by Richardson in 1866, and no doubt encouraged the further research for an effective and safe local anesthetic. It is within the memory of many of us when ethyl chloride was stocked in glass tubes ready for use. After breaking the fused tip of the glass vessel the ether was sprayed on the gum surrounding the tooth to be extracted until a coating of ice encircled the offending organ of mastication. The tooth was then extracted and the gum allowed to thaw. Needless to say that this method did not remain very long in general use, as it is doubtful which was the worse pain—the extraction of the tooth without the ether spray, or the thawing of the gum after the extraction. In my short experience of it there was no pain felt at the time of the extraction, if the gum was efficiently frozen, but many argue that the lack of feeling was due to temporary general anesthesia; this particular anesthetic being very quick in its action and subsequent recovery. The more reliable local anesthesia follows as a result of the introduction of cocaine in ocular practice by Roller in 1884 and by Drs. Hall and Halstead in dental practice in the same year. This drug, in turn, did not meet with much favor in this country as the effective strength, four per cent., was rather beyond safe limits. An important discovery was made by Corning in 1885, when he established that when the circulation of the blood was simultaneously interrupted, the action of cocaine was augmented. On account of this new knowledge it was found possible to employ smaller doses of cocaine—five-tenths to one per cent.—subcutanoeusly and still induce anesthesia in a few minutes. The circulation of the blood at this time was impeded by constriction of the tissues or the ether spray. It was only after the introduction of adrenalin in 1901 as a constituent of the local anesthetic solution that this method for extraction assumed its clinical importance: this drug having the peculiar effect previously stated on the circulation. From this time we are all well aware of the developments, both in its use and excessive abuse, which have taken place up to the present period. At a later date, 1905, another invaluable and less toxic drug was introduced; that is novocaine. It is less quick in its action, but a necessity in the surgery where local anesthesia is employed.

Local anesthesia, as its name implies, is the induction of insensibility to pain in a circumscribed area.

There are two methods employed, or a combination of both, according to the local area it is desired to render insensitive to pain. I refer to conduction anesthesia and infiltration anesthesia. The former, which consists of injecting over a main nerve in such a position, distant from the seat of operation, that the sensory branches supplying the tissues to be operated upon are rendered inactive. This method, according to our literature on the subject, has many advocates, but, as I have only had experience with it to a limited extent, in combination with the infiltration method, I do not purpose dealing with it in detail. I have come in contact with many patients in everyday practice who have submitted to this conduction method, and without exception they have suffered severe after pain. I refer to cases where a long needle is required. It would appear also that this latter operation is not without risks, as several cases of fracture of the needle are reported in the *Dental Cosmos*, April, 1919.

Its one favorable feature is that a very small amount of anesthetic agent is necessary through its being injected directly around a main nerve. The three common positions for this form of injection are between the internal and external oblique lines of the lower mandible, the mental foramen, and above the superior maxillary tuberosity.

In infiltration anesthesia the nerve endings beneath the surface of the tissues injected are rendered insensitive through their immediate proximity to the anesthetic solution used.

In dental practice this submucous injection is, where possible, most of all to be desired, but unfortunately the further distal is the tooth the more risk there is of this method failing until the third molar is approached. This infiltration method is more successful in the upper than in the lower jaw except for the lower incisor region. The advantages are that there is less disturbance of the normal condition and the circulation is more quickly induced to return, with the consequent diminution of unfavorable aftereffects. Generally speaking it is often better to keep to the infiltration anesthesia, even though a little pain be felt, which will sometimes happen in the molar region, rather than run the risk of recurrent pain in the conduction method. Indeed, even in the molar region, where the infiltration anesthesia is employed, the pain can be reduced to a minimum by waiting a little longer than usual, and allowing time for the anesthetic to penetrate.

The solutions which I am in the habit of using are locosthetic and novocaine.

Locosthetic is a convenient preparation of 0.75 per cent. cocaine with a suitable proportion of adrenalin chloride, stocked in a rubber-covered bottle, and always ready for use. Novocaine is prepared in tablets in which adrenalin is incorporated. The tablets are dissolved in a sterile solution, the amount of which is varied according to the strength required.

No special preparation of the patient is necessary, but about two hours after a meal is recognized as the best time for the operation. The patient should be assured that no pain will be felt, as there is a consciousness of something happening when the sockets are giving way.

Highly nervous people and children are not good subjects for local anesthesia.

### TYPE OF HYPODERMIC SYRINGE AND ITS STERILIZATION

There are several types of syringes in use according to the desires of the operator, the most useful types being the Fischer syringe which has a glass barrel and the all-metal syringe. For my part I use the all-metal syringe, with the heavy plunger and finger grips, and capable of containing twenty minims of locosthetic—one-seventh grain of cocaine.

To sterilize the syringe it should be taken and boiled each day after use, with the exception of the screw cap containing the leather washer, which latter should be placed in lysol, and have glycerine applied to the leather washer occasionally. After this procedure the syringe should be placed in the formalin cabinet, to be ready for use on the next occasion, when required. The needle should always be sharp and have the fine wire provided with it, dipped in lysol and inserted before boiling. It is a good plan to draw lysol into the syringe, press it out, and then draw in boiling water. It is no trouble, as both of these are always accessible.

### STERILIZATION OF THE MOUTH

Too much attention can not be given to this, as all aseptic precautions are strictly necessary and any neglect is bound to lead to trouble. Calculi should be removed and hydrogen peroxide applied to the gums. A mouth wash should be used and the tissues where the injection is to be made should be rubbed with dentalone or oil of cloves. In certain positions, where saliva is likely to collect, a wool roll will be found useful.

The next feature of importance to bear in mind is the area of sensory nerves. To induce local anesthesia, a clear comprehension of the course, position and distribution of the sensory nerves is necessary. In the upper jaw the innervation is more complicated than in the lower jaw. The upper derives its sensory nerves from the superior maxillary with its branches, the anterior and posterior dental and labial branches to mucous membrane of mouth.

The posterior dental branches pass downwards over tuberosity of superior maxillary just above the third molar tooth. In this position the anesthetic agent is introduced for conduction anesthesia in removal of upper molars. One branch from posterior dental runs to anterior dental and joins it opposite canine fossa. Filaments from these branches form a minute plexus in outer wall of superior maxillary above alveoli and send twigs to molar teeth and corresponding portion of gums. Others go to gums and mucous membrane of cheeks.

Anterior dental leaves superior maxillary just before its exit from the infra-orbital foramen; it enters special canal in antrum and communicates with posterior dental. In its course it gives off the middle dental to bicuspid teeth and other filaments to canine and incisor teeth. Labial branches descend beneath the levator labii to upper lip and mucous membrane of mouth.

The innervation of the lower jaw is not so complex. The inferior maxillary is the main sensory nerve and enters the inferior maxillary in the canal of that name. Branches from it form the inferior dental plexus from which proceed twigs to pulps and periosteum and others pierce bone to reach the gums. This nerve terminates through the mental foramen below the first or near the second lower bicuspid tooth, it innervates the skin over chin, the lower lip and its mucous membrane, the labial gums and anterior parts of periosteum. The lingual gums and the periosteum of this side are supplied by the lingual nerve.

### POSITIONS FOR INJECTIONS

Before proceeding to insert the needle it as as well to examine the mucous membrane around the offending tooth and determine the best situation for its introduction. The most successful cases promised are those in which a large sulcus is present, and in which more or less of the outlines of the roots are visible. In the case of the four upper incisors the needle should be inserted on the palatal side abount one-eighth of an inch from the gum margin on each side of the median line of the palate, and on the buccal side about the middle of the roots. This procedure will invariably result in complete anesthesia for the removal of the four upper incisors at one sitting. To extract the upper canine the injection is made on the palatal and buccal sides in a similar position to that for incisors. To inject for upper bicuspids, the needle is inserted on the palatal side, into the dense tissue without any difficulty and on the buccal side halfway up the roots with the needle parallel with the roots. In dealing with the upper molars the points for injection are relatively the same as for the bicuspids on the buccal and palatal sides. These latter teeth are more difficult to deal with, painlessly, than the other upper teeth, but I find that by injecting over the maxillary tuberosity a small quantity of solution and waiting a little longer, the pain felt, if any, is inconsiderable.

The point of introduction for the lower incisors and canines is a little below the gum margin on the labial and lingual surfaces. In the lower biscuspid region an injection on the lingual side to deal with the twigs from the lingual nerve and another just over the mental foramen below the first biscusipd tooth will result in complete anesthesia with the utmost confidence.

To inject for the first, second and third lower molars the syringe should be held horizontally on the labial sides and obliquely on the lingual sides at a point of entrance about one-eighth of an inch from the gum margin.

I consider these latter teeth the most unfavorable of all for infiltration anesthesia, and I have on many occasions employed the method of conduction anesthesia between the external and internal oblique lines as recommended by Fischer. I am no advocate for this method on account of the undesirable after-effects. The method as given by Fischer is as follows:

The patient's mouth being wide open the internal and external oblique lines are felt for. Between these two lines is a depression into the center of which the needle is introduced until bone is felt one c.m. above and one c.m. laterally to the molar surface of the lower teeth. A little injection on the lingual side of the molars to deal with the buccal nerve is also necessary.

Having extracted the tooth or teeth, certain after precautions are necessary. The sockets should be syringed with hot water and some sedative, such as dentalone—a combination of oil of cloves and chloretine introduced. Hot water should be held in the mouth for a few minutes to induce the return of the circulation as soon as possible and so decrease the liability to swelling. I always recommend patients to take a cup of hot coffee.—*The Dental Record.*

